# Economic Advantages of Telehealth and Virtual Health Practitioners: Return on Investment Analysis

**DOI:** 10.2196/15688

**Published:** 2020-05-21

**Authors:** Centaine L Snoswell, John B North, Liam J Caffery

**Affiliations:** 1 Centre for Online Health The University of Queensland Brisbane Australia; 2 Department of Orthopaedics The Princess Alexandra Hospital Brisbane Australia

**Keywords:** clinical services, e-health, health economics, health funding and financing, rural and remote health, workforce

## Abstract

**Background:**

Telehealth is a disruptive modality that challenges the traditional model of having a clinician or patient physically present for an appointment. The benefit is that it offers the opportunity to redesign the way services are offered. For instance, a virtual health practitioner can provide videoconference consultations while being located anywhere in the world that has internet. A virtual health practitioner also obviates the issues of attracting a specialist medical workforce to rural areas, and allows the rural health service to control the specialist services that they offer.

**Objective:**

The aim of this research was to evaluate the economic effects of 3 different models of care on rural and metropolitan hospital sites. The models of care examined were patient travel, telehealth using videoconferencing, and employment of a virtual health practitioner by a rural site.

**Methods:**

Using retrospective activity data for 3 years, a return on investment (ROI) analysis was undertaken from the perspective of a rural site and metropolitan partner site using a telehealth orthopedic fracture clinic as an example. Further analysis was conducted to calculate the number of patients that would be required to attend the clinic in each model of care for the sites to break even.

**Results:**

The only service model that resulted in a positive ROI for the rural site over the 3-year period was the virtual health practitioner model. The breakeven analysis demonstrated that the rural site required the lowest number of patients to recoup costs in the virtual health practitioner model of care. The rural site was unable to recoup its costs within the travel model due to the lack of opportunity for reimbursement for services and the requirement to cover the cost of travel for patients.

**Conclusions:**

Our model demonstrated that rural health care providers can increase their ROI by employing a virtual health practitioner.

## Introduction

Telehealth is a disruptive modality that challenges the traditional model which requires the clinician and patient to be physically present for an appointment. It is widely accepted that telehealth increases patient access, increases productivity potential for clinicians, and potentially reduces costs for service providers [1-4]. Although it is disruptive, telehealth often seeks to emulate traditional service models. For example, when a rural and remote service cannot provide specialist care, the patient is traditionally transported to a metropolitan partner facility; with telehealth, patients can access specialist care from the same metropolitan partner facility without having to travel.

Telehealth represents a valuable opportunity to redesign health service models in Australia. In one potential redesign, rural and remote health services can employ virtual health practitioners. A virtual health practitioner is an employee who works remotely but is otherwise considered to be a regular employee of the organization [5]. The standard telehealth model often functions by connecting two sites: one site employs a specialist health practitioner, and the other site requires a consultation from that specialist. The virtual health practitioner model enables the site that requires a specialist health practitioner to employ that staff resource directly. Use of telehealth by virtual health practitioners to provide specialist services has been previously reported; however, the economic advantages of telehealth for these sites have not been investigated [6].

Assuming regulatory requirements are met, a virtual health practitioner can be located at any site that has internet access, including metropolitan and rural areas. A virtual health practitioner can reduce patient travel and associated costs, which is of particular interest when travel is subsidized by the health care provider. Furthermore, employing virtual health practitioners can obviate the difficulty of attracting medical specialists to rural areas and can allow the health service employing the specialists to control their specialist workforce and the services they offer [7].

Using an orthopedic fracture clinic as an example, this research explores the economic impacts of 3 different models of care: telehealth using videoconferencing (rural site to metropolitan partner), patient travel (rural site to metropolitan partner), and employing a virtual health practitioner at a rural site. The aim of this study was to evaluate the costs and ROI for rural and metropolitan sites for each of the 3 models of care.

## Methods

An analysis of return on investment (ROI) was undertaken from the perspectives of a rural site and a metropolitan partner site. Ethics approval was obtained from the Metro South Human Research Ethics Committee, HREC/17/QPAH/438.

### Setting

The state health department in the Australian state of Queensland is divided into 16 hospital and health services (HHSs). Some of these HHSs are located in metropolitan areas and provide a wide range of specialist services. In addition, some HHSs are located in rural and remote areas, where recruitment and retention of health care professionals can be difficult [2]. Patients are transferred to a metropolitan HHS when the rural HHS cannot provide specialty care.

This example is based on a consultant-led fracture clinic using real-time video consultations between a tertiary facility, Princess Alexandra Hospital, which is located in metropolitan Brisbane (the capital city of Queensland), and Mount Isa Hospital, which is located in remote Queensland. A pilot study examining the cost-effectiveness of this clinic demonstrated substantial cost savings for the remote HHS [8,9]. Prior to the introduction of telehealth, fracture clinic patients were required to drive or be transported to Townsville Hospital (approximately 900 kilometers from Mount Isa Hospital).

Mount Isa Hospital is part of the North West HHS, which spent approximately A$16.6 million on patient transport in the 2016-2017 financial year, accounting for 9.4% of their spending [10]. Since only a small proportion of patients (17% in the 2016-2017 financial year) received a subsidy for their travel, these costs do not represent the full societal burden of patient travel for health care services. The Queensland Health Travel Subsidy Scheme eligibility criteria now state that individuals are only eligible for subsidized travel if they are “unable to use telehealth to access the required eligible specialist medical service” [11].

### Data Collection

Retrospective data for the Metro South HHS telehealth orthopedic fracture clinic for the financial years of 2014-2015, 2015-2016, and 2016-2017 were accessed from the hospital data repository (Table 1).

**Table 1:** Telehealth activity reported for the orthopedic clinic during the 2014-2015, 2015-2016, and 2016-2017 financial years.

Financial year	Total patients, n	Clinics, n	New patient bookings, n (%)	Review patient bookings, n (%)	Adult patient bookings, n (%)	Pediatric patient bookings, n (%)	Failed to attend, n (%)
2014-2015	321	31	175 (55)	146 (45)	279 (87)	42 (13)	53 (17)
2015-2016	1235	82	686 (56)	549 (44)	776 (63)	459 (37)	321 (26)
2016-2017	1136	82	612 (54)	524 (46)	915 (81)	221 (19)	318 (28)

Clinic attendance information was used to calculate the costs and ROIs of three different care models: a videoconference telehealth model (the patient at Mount Isa Hospital contacts a specialist at Princess Alexandra Hospital), a travel model (the patient travels to Townsville Hospital for a telehealth consultation), and a virtual health practitioner model (the patient at Mount Isa Hospital contacts a remote virtual practitioner employed by Mount Isa Hospital). The involved hospitals, descriptions of the service models, costs, and income for each care model are outlined in Table 2.

**Table 2:** Details of the three examined models of care.

Characteristic	Telehealth clinic	Patient travel	Virtual health practitioner
Involved hospitals	Mount Isa Hospital (small remote hospital) and Princess Alexandra Hospital (tertiary metropolitan hospital)	Townsville Hospital (large regional hospital)	Mount Isa Hospital (small remote hospital)
Service model description	A videoconference is held between the patient at Mount Isa Hospital and a specialist at Princess Alexandra Hospital. The specialist is employed by Princess Alexandra Hospital.	Patients travel from their home to receive in-person care at Townsville Hospital, which is the nearest hospital that provides orthopedic services.	Mount Isa Hospital directly employs a specialist to conduct videoconference consultations with patients located at Mount Isa Hospital. The specialist is located in a different geographical location from Mount Isa.
Cost allocation	Mount Isa Hospital pays for local staff (eg, nurse, resident medical officer) and also pays Princess Alexandra Hospital for the specialist and administration staff time. Princess Alexandra Hospital pays for the clinical assistant for the specialist and for the remaining administration time.	Townsville Hospital pays for local staff as normal. Mount Isa Hospital pays to subsidize patient travel for individuals who claim from the Queensland Health Patient Travel Subsidy Scheme.	Mount Isa Hospital pays for all costs.
Income	Princess Alexandra Hospital claims activity-based funding reimbursement as the telehealth provider. Mount Isa Hospital claims activity-based funding reimbursement as the telehealth recipient.	Townsville Hospital claims activity-based funding reimbursement for the appointment as the consultation provider.	Mount Isa Hospital claims activity-based funding reimbursement for the appointment as the telehealth provider.

### Cost Analysis

In ROI analysis, the cost-to-benefit ratio is calculated by dividing the total net benefit by the total cost, allowing outcomes to be expressed in terms of percentage of gain relative to cost [12]. To calculate the ROI in this study, the total cost was based on the costs of human resources and patient-subsidized travel, and the net benefit was determined from the activity-based funding each site received for each nonadmitted outpatient event.

All human resource costs were calculated using published wages for the 2016-2017 financial year [13], and on-costs were added according to the workplace agreements (23% extra for medical officers and 29% extra for all other staff). To calculate wages for previous years, a discount rate of 2.5% per year was used in accordance with Queensland Health workplace agreements. Income for each site was calculated by assuming that 100% of patients who attended appointments received the applicable activity-based funding. Activity-based funding rebates were based on the appropriate National Weighted Activity Unit (NWAU) code for the respective years, taking into account whether the event was a new or review case [14]. NWAU 20.29 was claimed by the provider site where the consultant was located, while NWAU 40.16 was claimed by the provider site where the patient and support clinical staff were present. NWAU funding rates do not discriminate between new and review appointments, unlike the Queensland Weighted Activity Unit (QWAU) values, which were unavailable [15]. Failure to attend (FTA) rates were assumed to be the same across the 3 models. All prices are reported in Australian dollars (US $0.62) and have not been converted to 2018 prices, as they represent the cumulative economic implications for a 3-year period.

Travel subsidy costs were calculated assuming that 17% of the non-FTA population for each financial year received subsidized travel. Although the majority of patients are eligible to claim the travel subsidy, very few take advantage of the subsidy. The selection of 17% was based on the 2016-2017 annual report from the North West HHS, in which Mount Isa Hospital is situated [16]. An average travel cost of A$1447 per individual receiving the subsidy was assumed based on the 2016-2017 annual report, which stated that A$14.48 million was provided in patient travel subsidies to support 8623 patients. The travel amount was discounted by 2.5% per year for the two prior financial years, in accordance with local policies [16].

### Breakeven Point

Using the calculations of cost per clinic, it is possible to calculate the breakeven point (ie, the minimum number of patients required per clinic to cover the cost of the service provision for that site). The breakeven point is the point at which the cost of running the outpateint clinic is negated by the income received from the appointments conducted. As FTA appointments do not yield income, they do not count toward the number of appointments required to break even.

### Sensitivity Analysis

To investigate the uncertainty, we performed a sensitivity analysis. The income for each site was recalculated assuming 10% and 35% FTA rates. To investigate the effects of travel reimbursement, the population with subsidized travel was increased from 17% to a hypothetical 25%. Additionally, using the base case figures, we calculated the number of appointments required for each clinic to break even and cover its costs.

## Results

### Cost Analysis

Given the costs for providing each model of service, a cumulative 3-year net benefit was calculated for each site (Table 3). The analysis demonstrated that the only service model that resulted in a positive ROI for the rural site was the virtual health practitioner model of care.

The highest net benefit for the rural Mount Isa site was demonstrated for the virtual health practitioner model, followed by the telehealth model (Table 4). The benefits were higher compared to the patient travel model, where the site bears all the costs and does not generate any income. If patient travel reimbursement is increased from the assumed 17% of travel costs to 25%, the travel costs for this single outpatient clinic are approximately A$1 million.

**Table 3:** Human resource costs associated with the 3 models of care at each clinic.

Site and staff role			Hourly rate, A$			On-cost			Hours required per clinic			Cost, A$		
**Telehealth clinic**														
	**Princess Alexandra Hospital**													
		Radiographer		50.61			0.29			4			261.13	
		Administration (organization and clinic)		34.88			0.29			2			89.99	
		Total												351.12
	**Mount Isa Hospital**													
		Orthopedic specialist		134.57			0.23			4			662.08	
		RMO^a^		59.35			0.23			4			292.00	
		Nurse		39.20			0.29			4			202.25	
		Administration (organization and clinic) + 2 hours paid to Princess Alexandra Hospital		34.88			0.29			10			449.96	
		Plaster technician		28.73			0.29			4			148.23	
		Total												1754.52
**Patient travel**														
	**Townsville Hospital**													
		Orthopedic specialist		134.57			0.23			4			662.08	
		Radiographer		50.61			0.29			4			261.13	
		Administration (organization and clinic)		34.88			0.29			6			269.98	
		Plaster technician		28.73			0.29			4			148.23	
		Total												1341.42
	**Mount Isa Hospital**													
		Travel subsidy for patients		N/A^b^			N/A			Varied			Varied	
**Virtual health practitioner**														
	**Mount Isa Hospital**													
		Orthopedic specialist (off site)		134.57			0.23			4			662.08	
		RMO		59.35			0.23			4			292.00	
		Radiographer		50.61			0.29			4			261.13	
		Nurse		39.20			0.29			4			202.25	
		Administration (organization and clinic)		34.88			0.29			8			359.97	
		Plaster technician		28.73			0.29			4			148.23	
		Total												1925.66

^a^RMO: resident medical officer.

^b^Not applicable.

**Table 4:** Three-year ROI analysis for the three service models. All values are given in Australian dollars.

Clinic service model and site		2014-15				2015-16				2016-17				Three-year total				Three-year net benefit (profit)	
		Cost		Income (FTA^a^ 10%-35%)		Cost		Income (FTA 10%-35%)		Cost		Income (FTA 10%-35%)		Cost		Income (FTA 10%-35%)			
**Telehealth clinic**																			
	Mount Isa Hospital referral site		50,507		17,847 (13,989-19,369)		140,361		153,693 (134,986-186,904)		143,870		121,244 (109,446-151,540)		334,738		292,785 (258,421-357,813)		–41,954
	Princess Alexandra Hospital provider site		10,108		67,547 (52,589-72,815)		28,090		234,199 (205,693-284,806)		28,792		184,082 (166,169-230,080)		66,990		485,828 (424,450-587,701)		418,839
**Patient travel**																			
	Mount Isa Hospital referral site^b^		–75,158 (–110,526)		N/A^c^		–296,388 (–425,234)		N/A		–279,445 (–391,146)		N/A		–650,991 (–926,906)		N/A		–650,991 (–926,906)
	Townsville Hospital provider site		39,580		61,488 (52,589-72,815)		107,314		240,503 (205,693-284,806)		109,996		194,289 (166,169-230,080)		256,890		496,281 (424,450-587,701)		239,390
**Virtual health practitioner**																			
	Mount Isa Hospital referral site		55,433		67,547 (52,589-72,815)		154,053		234,199 (205,693-284,806)		157,904		184,082 (166,169-230,080)		367,390		485,828 (424,450-587,701)		118,439

^a^FTA: failure to attend.

^b^Costs for patient travel for this site are represented as cost (25% patient travel paid).

^c^Not applicable.

The analysis demonstrated that the only service model that resulted in a positive ROI for the rural site over the 3-year period was the virtual health practitioner model. The ROI for the rural site was –100% for the patient travel model, from which they derived no income, –12.5% for the telehealth model, and 32% for the virtual health practitioner model. Moreover, the ROIs for the metropolitan site were 93% for patient travel and 625% for telehealth; because the virtual health practitioner model is not applicable to the metropolitan site, it incurred neither cost nor income.

### Breakeven Point for Each Model of Care

**Figure 1:**
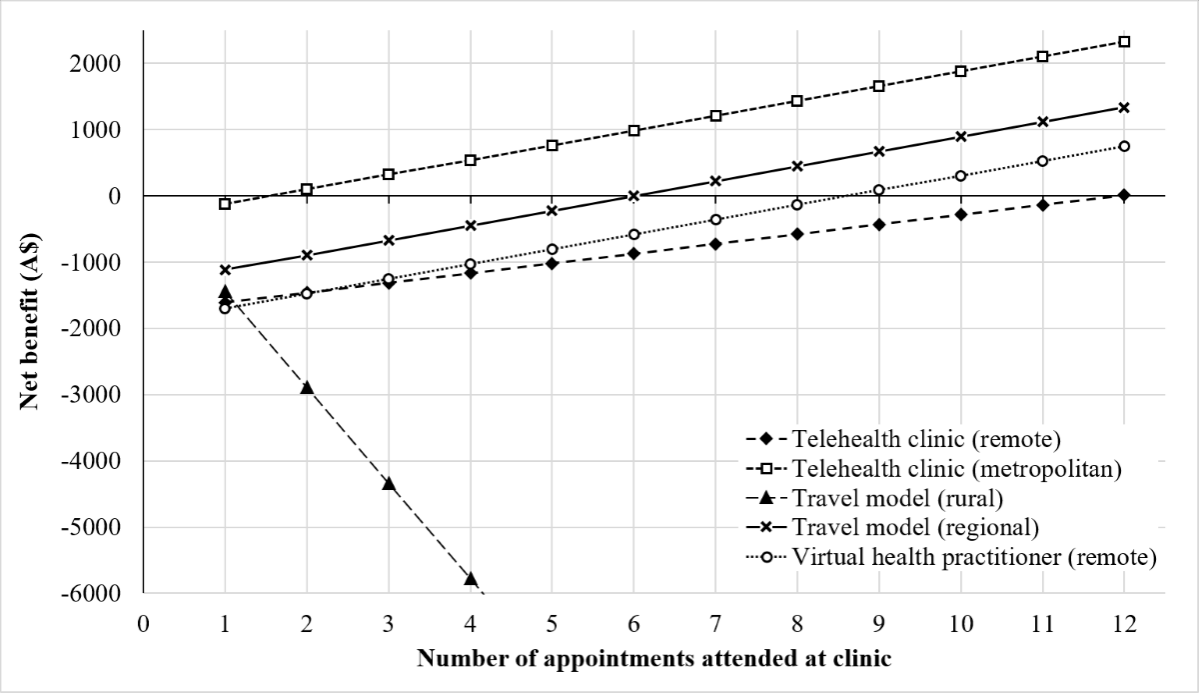
The breakeven point for each model of care.

The breakeven analysis demonstrated the number of appointments that each site needs to conduct in order to cover the costs of providing the clinic service (Figure 1). For the travel model of care, the provider site must complete a minimum of 6 appointments to cover their costs; however, the rural site is unable to recoup their costs within this model due to the cost of travel and lack of income opportunity. Alternately, in the telehealth model of care, the provider site can break even by providing a minimum of 2 appointments, while the rural site must provide a minimum of 12 appointments. This disparity between the number of patients required to break even is due in part to the cost sharing arrangements for the service being modelled, where the rural site covers some human resource costs for the provider site (health practitioner and administration).

## Discussion

### Principal Findings

Our model demonstrated that rural health care providers can increase their ROI if they employ a virtual health practitioner as an alternative to subsidized patient travel or if they refer patients to telehealth clinics provided by a tertiary center. This largely results from savings from patient travel subsidies and generation of activity-based funding under the virtual health practitioner model. Increasing this to include all eligible patients (100%) would increase the volume of negative ROI for the model (Figure 1) but would not change the results. Further, our modelling showed that the rural site could break even when 3 patients attended, rather than 12. Additionally, we demonstrated that rural sites receive greater net benefits from using a virtual health practitioner than from the other models of care. The greater the economic benefit that is achieved by the rural sites, the greater the benefit to the community in which they are located.

Previous studies have shown that in the context of the Australian health care system, rural sites and metropolitan sites can gain economic benefit from implementing a telehealth service model [2-4]. International studies have also demonstrated cost mimimization potential for videoconferencing in orthopedic applications [17,18] and high acceptance from rural health care practitioners and patients when specialists provide services using videoconferencing [19,20]. Our study adds to the body of knowledge on telehealth economics by modelling the use of virtual health practitioners; to the best of our knowledge, this has not been done previously.

In addition to economic advantages, the virtual health practitioner model may provide other benefits. Often, it is difficult to entice specialist clinicians to move to rural and remote areas to provide services [2,6]. The virtual health practitioner model provides an alternative by which rural and remote hospitals can gain specialty services for positions for which they are unable to recruit or retain staff or for positions that only require a small fraction of a full-time equivalent. One additional benefit is that patients can be referred back to primary care sooner, if appropriate, which will support the local rural health workforce.

### Implications for Practice

While the economic focus in this paper is the optimization of outpatient clinics, the aim is not to advocate for a purely virtual care model for Australian patients. Instead, as models of care change to integrate telehealth and other virtual care provision modalities, we propose that alternate funding and employment models to those used in traditional in-person models of care (and telehealth emulations of these models) should be possible. Patients will still be required to travel for procedures, diagnostics, and outpatient appointments where a telehealth consultation is not appropriate.

Additional alternative models of care may present economic advantages; for example, a store-and-forward consultation with feedback to the general practitioner may be sufficient to diagnose and treat a simple fracture [21]. As telehealth services mature, patient cases can ideally be triaged to the most appropriate service model for their condition.

### Strengths and Limitations

A strength of this study is the use of activity data from an existing telehealth service. By basing our analysis calculations on actual activity, we were able to present realistic economic examples for the 3 service models.

A limitation of this study is that the economic analysis is based on a specific orthopedic clinic example; therefore, the findings lack generalizability. The ROI was estimated within the public funding models for the Australian state of Queensland (activity-based funding and travel subsidy scheme) and was based on local transportation costs. The model would require adaptation if it were transferred to alternate contexts.

The economic analysis presented here was for a service which experiences high and regular activity; the ROI estimates would need to be recalculated if the analysis were adapted for a service with low activity. As demonstrated by the example of this orthopedic fracture clinic, the virtual health practitioner model has a lower patient attendance rate requirement to break even on clinic costs. Additionally, the proportion of the population who claim the travel subsidy for this analysis was assumed to be 17%. If this percentage was increased to reflect the near-100% eligibilty of the population, it would only serve to reduce the already negative ROI for the small rural site; for this reason, a pragmatic assumption was made to reflect the real-world scenario.

The substitution rate of telehealth for in-person encounters is an additional variable that influences the ROI. The teleorthopedic service for the fracture clinic described in this paper is highly amenable to telehealth because physical examination of the patient is largely mitigated by the supplementary information provided by x-rays. When a physical examination is required, it can be performed by a junior doctor at the rural site. For other services, such as a general orthopedic clinic, it may not be possible to provide consultations by telehealth; as a result, the substitution rates will be lower. Different medical specialities have different telehealth substitution rates [22]. Hence, the economic findings of this study cannot be extended to all specialities. Economic modelling of blended models involving care delivered by a combination of telehealth, virtual health practitioners, outreach, and patient travel is an area for future research.

## Abbreviations

FTA: failure to attend
NWAU: National Weighted Activity Unit
RMO: resident medical officer
ROI: return on investment
